# Homogeneous crystalline FeSi_2_ films of c (4 × 8) phase grown on Si (111) by reactive deposition epitaxy

**DOI:** 10.1186/1556-276X-8-510

**Published:** 2013-12-05

**Authors:** Zhi-Qiang Zou, Li-Min Sun, Gao-Ming Shi, Xiao-Yong Liu, Xu Li

**Affiliations:** 1 Center for Analysis and Testing, Shanghai Jiao Tong University, 800 Dongchuan Road, Shanghai 200240, China; 2 Department of Physics, Shanghai Jiao Tong University, 800 Dongchuan Road, Shanghai 200240, China

**Keywords:** Iron silicides, Reactive deposition epitaxy, Thin films, Scanning tunneling spectroscopy, X-ray photoelectron spectroscopy

## Abstract

The growth of iron silicides on Si (111) using reactive deposition epitaxy method was studied by scanning tunneling microscopy and X-ray photoelectron spectroscopy (XPS). Instead of the mixture of different silicide phases, a homogeneous crystalline film of *c* (4 × 8) phase was formed on the Si (111) surface at approximately 750°C. Scanning tunneling spectra show that the film exhibits a semiconducting character with a band gap of approximately 0.85 eV. Compared with elemental Fe, the Fe 2*p* peaks of the film exhibit a lower spin-orbit splitting (−0.3 eV) and the Fe 2*p*_3/2_ level has a smaller full-width at half maximum (−0.6 eV) and a higher binding energy (+0.3 eV). Quantitative XPS analysis shows that the *c* (4 × 8) phase is in the FeSi_2_ stoichiometry regime. The *c* (4 × 8) pattern could result from the ordered arrangement of defects of Fe vacancies in the buried Fe layers.

## Background

Iron silicides grown on silicon surfaces have attracted much attention in the last decade because of their possible applications in different technological areas [1-4]. The equilibrium Fe-Si phase diagram shows that there exist four stable bulk compounds: Fe_3_Si crystallizing in cubic *D*0_3_ structure, simple cubic ϵ-FeSi, tetragonal *α*-FeSi_2_, and orthorhombic *β*-FeSi_2_[5].These iron silicides exhibit metallic, semiconductor, or insulating behavior depending on their structures. For example, Fe_3_Si is ferromagnetic and is a promising candidate as spin injectors in future spintronic devices such as magnetic tunnel junctions [6]. *β*-FeSi_2_ is semiconducting with a direct band gap of approximately 0.85 eV, which fits into the window of maximum transmission of optical fibers and is expected to be a suitable material for optoelectronic devices such as light detectors or near-infrared sources [2,7]. In addition to the above stable compounds, metastable iron silicide phases which do not exist in the bulk phase diagram can also be grown and stabilized on silicon surfaces by epitaxy due to the enhanced surface energy of thin films. It has been reported that very thin films of metastable γ-FeSi_2_ phase with a cubic CaF_2_ structure [8,9], FeSi_1+*x*_ (0 ≤ *x* ≤ 1) phase with a defect CsCl structure [10,11] and a new silicide phase with a *c* (4 × 8) surface periodicity [2,12,13] can be grown on Si (111) substrate by solid-phase epitaxy (SPE), which was realized by depositing iron on the silicon substrate at room temperature and then annealing the film at an elevated temperature.

Despite the interesting properties and potential applications, it is challenging to control the silicide reaction at the Fe/Si interface and grow a flat and single-phase thin film of iron silicide with the demanded structure. Due to the variety of existing compounds and the complexity of growth kinetics, the iron silicide thin films usually grow into a mixture of different phases with heterogeneous morphology [2,5,13]. Different from the silicide reaction in SPE, which is realized under iron-rich condition, reactive deposition epitaxy (RDE) (deposition of iron on the silicon substrate heated to a determined temperature) most probably involves diffusion of monomers on the surface, which may lead to the formation of unusual silicide structures. It has been reported that RDE favors the production of Si-rich phases and single crystalline epitaxial structures [14,15]. In this paper, we performed a scanning tunneling microscope (STM) study on the reactive epitaxial growth of iron silicides on Si (111)-(7 × 7) surface at different temperatures. We found that a thicker homogeneous and crystalline *c* (4 × 8) iron silicide thin film can be formed on the Si (111) surface with an extremely low deposition rate. The thickness of the film can be up to approximately 6.3 Å, which is significantly larger than that obtained previously by RDE method. This film could be used in the optoelectronic devices or serve as a precursor surface applicable in magnetic technological fields.

## Methods

Iron silicide thin films were grown on Si (111) substrates by using an ultrahigh vacuum (UHV) molecular beam epitaxy-STM system (Multiprobe XP, Omicron, Taunusstein, Germany) with a base pressure of less than 5.0 × 10^−11^ mbar. P-doped, n-type Si (111) substrates with resistivity of approximately 1 Ω cm were cleaned in UHV by the well-established annealing and flashing procedures [16]. Iron was deposited on the clean substrates by heating iron lumps (purity 99.998%) in a Mo crucible with electron bombardment. The iron flux was monitored by an internal ion collector mounted near the evaporation source. During deposition, the substrates were heated by direct current and the temperatures were measured using an infrared pyrometer. The deposition rate was controlled from approximately 0.01 to 0.07 ML min^−1^ (1 ML *=* 1 iron atom per 1 × 1 surface mesh = 7.8 × 10^14^ atoms cm^−2^) [13]. An electrochemically etched tungsten tip was used for scanning. All STM images were recorded at room temperature with a bias voltage (*V*_s_) of approximately 2.0 V and a tunneling current (*I*) of 0.1 to 0.25 nA.

X-ray photoelectron spectroscopy (XPS) spectra were acquired with a Kratos Axis Ultra DLD spectrometer using a monochromatic Al Kα source (1,486.6 eV). A detailed description of the experimental apparatus and the measurement conditions can be found in [17]. The XPS peak areas and peak decompositions (i.e., curve fitting) were determined using software XPSPEAK 4.1 [18]. Prior to fitting, Shirley background was subtracted and then peaks were fitted with mixed Lorentzian-Gaussian functions. The spectra were deconvoluted into components consisting of spin-orbit split Voigt functions [the intensity of the (Fe, Si) 2*p*_1/2_ is half that of the (Fe, Si) 2*p*_3/2_, and the full-width at half maximum (FWHM) is the same for both the splitting peaks]. The smallest number of components, with which a good fitting can be achieved for the experimental data, was adopted for the chemical state analysis.

## Results and discussion

**Figure 1:**
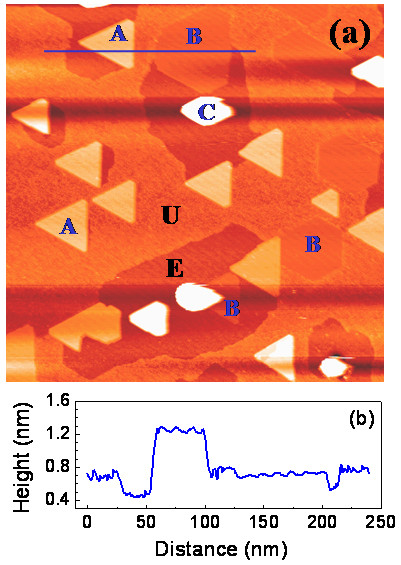
**STM image of the typical silicide islands and line profile showing the heights of A and B islands. (a)** STM image (400 × 400 nm^2^; *V*_s_ = 2.0 V; *I* = 0.15 nA) of the typical silicide islands grown at 650°C by depositing 1 ML of Fe on the Si (111) surface. E and U represent the etched region and unetched region, respectively. Three types of islands are observed. A and B are the tabular islands grown above and below the unetched Si-adatom layer, respectively. C is the three-dimensional islands. Most of the A islands exhibit an equilateral-triangle shape. **(b)** The line profile along the line in **(a)** shows that the heights of A and B islands with respect to the etched surface region are approximately 7.9 and 1.9 Å, respectively.

Similar to the SPE, the growth temperature of the RDE also has an important influence on the crystal structures of the iron silicides. When the growth temperature is below approximately 650°C, a mixture of different iron silicide phases with heterogeneous morphology develops on the Si (111) surface. Figure1a shows a STM image of the typical silicide islands grown at 650°C by depositing 1 ML of Fe on the Si (111) surface with a deposition rate of 0.015 ML min^−1^. It can be seen that after silicide reaction, the Si substrate surface can be divided into two regions: the etched silicon layer (region E) and the unetched silicon layer (region U). The step height between these two regions is approximately 3.1 Å. Both the region E and region U appear to be (1 × 1) silicon covered by a ‘sea’ of Si adatoms. The iron silicide islands can be categorized into three types. Type A is the tabular islands with a height of approximately 4.8 Å above the unetched Si-adatom layer (approximately 7.9 Å above the etched Si layer), as shown in the height profile taken along the line across the silicide islands and Si terraces (Figure 1b). This value is the multiples of 1.57 Å, the half of the bulk Si (111) spacing. Most of the type A islands exhibit an equilateral-triangle shape with edges oriented along the Si < −110 > directions, coinciding with the threefold symmetry of the Si (111) substrate. Type B islands are also tabular and grow approximately 1.9 Å above the etched surface regions. The third type of islands (type C) is three-dimensional (3D) and has a height more than 83.0 Å from the etched Si layer.

**Figure 2:**
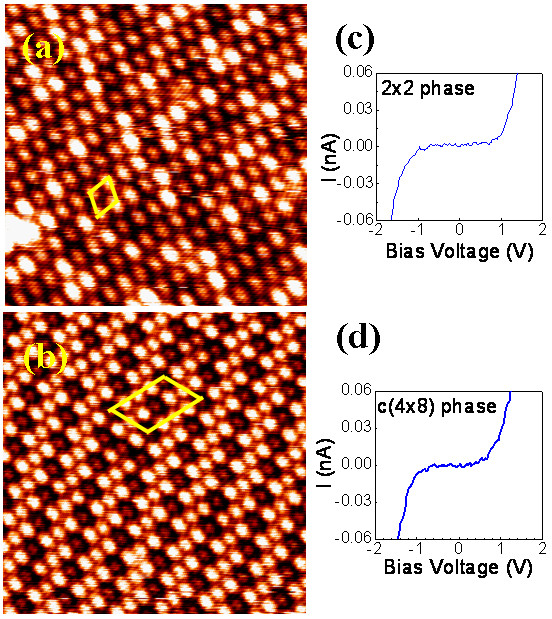
**STM images and scanning tunneling spectra for types A and B islands. (a)** High-resolution STM image (10 × 10 nm^2^; *V*_s_ = 2.0 V; *I* = 0.25 nA) of the surface of type A islands. A rhombic unit cell showing the (2 × 2) reconstruction is outlined. **(b)** High-resolution STM image (10 × 10 nm^2^; *V*_s_ = 2.0 V; *I* = 0.15 nA) of the surface of type B islands. A parallelogram unit cell showing the *c* (4 × 8) reconstruction is outlined. **(c,d)** Scanning tunneling spectra measured on types A and B islands, respectively, showing semiconducting characteristics with a band gap of approximately 0.85 to 0.9 eV.

Figure 2a,b shows the high-resolution images of the type A and type B islands, respectively. It can be seen that the surface of type A islands exhibits a hexagonal closed-packed symmetry with a (2 × 2) periodicity. Due to the lower surface energy of Si, the metal-silicon compounds are generally terminated by one or two Si layers. Thus, the 2 × 2 reconstruction on the iron silicides is due to the Si adatom ordering [19]. Similar to the type A islands, the type B islands also exhibit a (2 × 2) surface periodicity. However, two types of protrusion, bright and dark, are observed and they are ordered in a *c* (4 × 8) network. Since the contrast of bright and dark protrusions in the STM images is dramatically changed with the amplitude or the sign of the sample voltage, the *c* (4 × 8) periodicity is expected to have a pronounced spectroscopic origin. As the silicide is terminated by a pure Si top layer, this effect could arise only from the underlying Fe or Si layers of the silicide.

**Figure 3:**
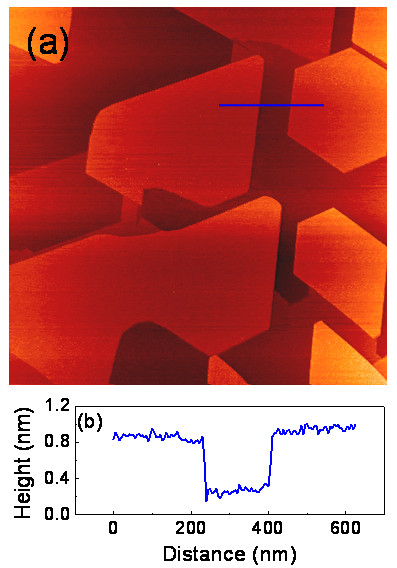
**STM image of the homogeneous *c *(4 × 8) iron silicide thin film and line profile. (a)** STM image (2,000 × 2,000 nm^2^; *V*_s_ = 2.0 V; *I* = 0.2 nA) of the homogeneous *c* (4 × 8) iron silicide phase grown at 750°C by depositing 1.5 ML of Fe on the Si (111) surface. The largest area of the *c* (4 × 8) tabular island is up to approximately 1.0 μm^2^. **(b)** The line profile along the line in **(a)** shows that the height of the *c* (4 × 8) tabular islands is approximately 6.3 Å with respect to the substrate terrace.

With the increase of growth temperature, the tabular islands become enlarged and cover more area of the substrate surface, whereas the number density of the 3D islands (i.e., type C islands) decreases. Figure 3a shows a STM image of the silicide islands grown at approximately 750°C by depositing 1.5 ML of Fe on the Si (111) surface. It can be seen that the substrate surface is almost covered by the tabular islands and no 3D islands are observed. The average size of the tabular islands rises to approximately 600 nm in diameter. The shape of the tabular islands changes from equilateral triangle to polygon, and some islands are connected to each other. However, the edges of the polygonal islands are still kept in the Si < −110 > directions. The high-resolution STM images show that all these tabular islands have the *c* (4 × 8) surface structure, indicating that they are type B islands. The type B islands are the only iron silicide phase formed on the Si substrate at approximately 750°C. The cross-sectional profiles show that the polygonal islands have the same height of approximately 6.3 Å, as shown by Figure 3b. This result is different from previous results obtained by means of SPE. Within the SPE technique, the well-ordered *c* (4 × 8) structure can be formed only at a Fe exposure lower than 1.5 ML and after high temperature annealing at about 600°C. The *c* (4 × 8) silicide phase exists only in the ultrathin film regime with a definite thickness in the range of 1.4 to 1.9 Å. If the Fe coverage is above 1.5 ML, a different type of silicide, namely, the (2 × 2) phase will grow into islands on top of the *c* (4 × 8) film [2]. This phenomenon could be attributed to the iron-rich environment of SPE because the *c* (4 × 8) phase is reported to have a FeSi_2_ stoichiometry and the Si atoms diffused to the reaction sites are insufficient [2]. The single *c* (4 × 8) phase and the larger thickness of the *c* (4 × 8) film obtained by the RDE method can be attributed to the supply of sufficient free Si atoms during the silicide reaction.

Previous studies showed that several metastable silicides [1 × 1, 2 × 2, and *c* (4 × 8) phases] that do not exist in the bulk phase diagram can be grown epitaxially on the Si (111) substrate under the strain from the substrate. The 1 × 1 phase can be assigned to the FeSi with a CsCl structure, while the 2 × 2 phase can be assigned to the γ-FeSi_2_ with a CaF_2_ structure and the FeSi_1 + *x*_ (0 ≤ *x* ≤1) with a defect CsCl structure [4]. The FeSi_1 + *x*_ (0 ≤ *x* ≤1) can be derived from the CsCl structure by introducing Fe vacancies distributed in a random fashion. The heights observed for the type A islands prove that the 2 × 2 phase is FeSi_1 + *x*_ (0 ≤ *x* ≤1) because the corresponding crystal structure has a spacing of 1.57 Å between equivalent atomic planes. If the 2 × 2 phase is γ-FeSi_2_ in the CaF_2_ structure, the heights in multiples of 3.14 Å should be observed [8,10]. Furthermore, the tunneling current–voltage (*I-V*) curve measured on top of the type A islands (Figure 2c) exhibits a semiconducting character with a band gap of approximately 0.9 eV, verifying that the 2 × 2 phase is not γ-FeSi_2_ because γ-FeSi_2_ is metallic [5,9]. The *c* (4 × 8) pattern could result from the formation of periodic defects of vacancies and/or Si substitution on the Fe sites in the buried Fe layers. These defects modify the local density of states above the Si atoms of the topmost layer, resulting in the different brightness of the protrusions [2,13]. Similar to the 2 × 2 phase, the *I-V* curve measured on top of the *c* (4 × 8) structure also shows a semiconducting character with a band gap of approximately 0.85 eV, as shown in Figure 2d.

**Figure 4:**
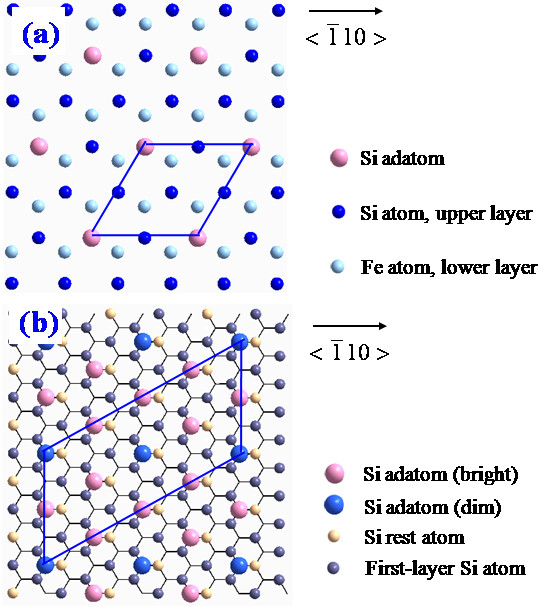
**Top views of the structure models for iron silicides. (a)** The 2 × 2 structure model. **(b)** The *c* (4 × 8) structure model proposed by Krause et al. [2,12]. A rhombic unit cell and a parallelogram unit cell are outlined in **(a)** and **(b)**, respectively.

Figure 4a shows the top view of the 2 × 2 structure model and a rhombic unit cell is outlined. The 2 × 2 superstructure was formed by the relaxation of three Si atoms towards the Fe layer and a Si adatom resides on the H_3_ site. The *c* (4 × 8) structure model proposed by Krause et al. [2,12] is shown in Figure 4b and a parallelogram unit cell is also outlined. This model is based on the CsCl-type structure in which the ordered periodic vacancies of Fe atoms exist under the Si adatom layer. The Si adatoms occupy the T_4_ positions, i.e., directly above the Fe sites of the second film layer.

**Figure 5:**
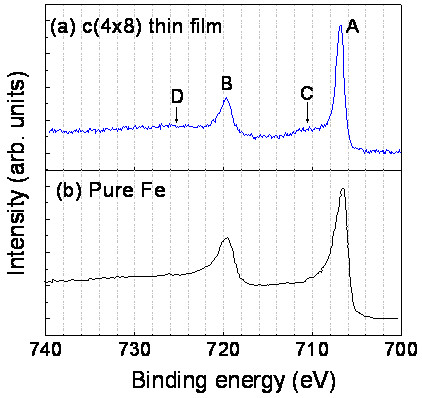
**Comparison of the XPS Fe 2*p *spectra for the *c *(4 × 8) thin film and pure Fe. (a)** XPS Fe 2*p* spectrum for the *c* (4 × 8) thin film grown on the Si (111) substrate. **(b)** XPS Fe 2*p* spectrum of pure Fe taken from [20].

In order to obtain further insight into the chemical state of the *c* (4 × 8) phase, we performed an XPS study on the *c* (4 × 8) thin film grown on the Si (111) substrate at approximately 750°C. Figure 5a shows the XPS spectrum measured near the Fe 2*p* peak. For comparison, the spectrum for clean Fe is also reproduced from [20] in Figure 5b. The binding energies of the Fe 2*p*_3/2_ peak (label A) and Fe 2*p*_1/2_ peak (label B) for the *c* (4 × 8) phase are 706.8 and 719.7 eV, respectively. The broad and weak peaks C (approximately 708 to 714 eV) and D (approximately 722 to 729 eV) appearing at the higher energy sides of A and B, respectively, correspond to the Fe 2*p* doublet of the Fe oxide phase, indicating that the iron silicide was partly oxidized during the sample transfer process. Compared with elemental Fe, the Fe 2*p* peaks of the *c* (4 × 8) film exhibit a lower spin-orbit splitting (−0.3 eV). The Fe 2*p*_3/2_ peak of the *c* (4 × 8) film has a smaller FWHM (−0.6 eV) and a higher binding energy (+0.3 eV). The latter two values are close to those (−0.55 and +0.4 eV, respectively) reported for the FeSi_2_ phase by Egert and Panzner [21]. The decrease of the Fe 2*p*_3/2_ FWHM can be interpreted from the aspect of crystallographic structure of the iron silicide. Crystallographic data show that from pure Fe to FeSi_2_, the interaction of adjacent Fe atoms decreases because the coordination number of the Fe nearest neighbors becomes less and their mutual distance grows. The Fe 2*p*_3/2_ line shape of FeSi_2_ shows a more atomic-like character.

**Figure 6:**
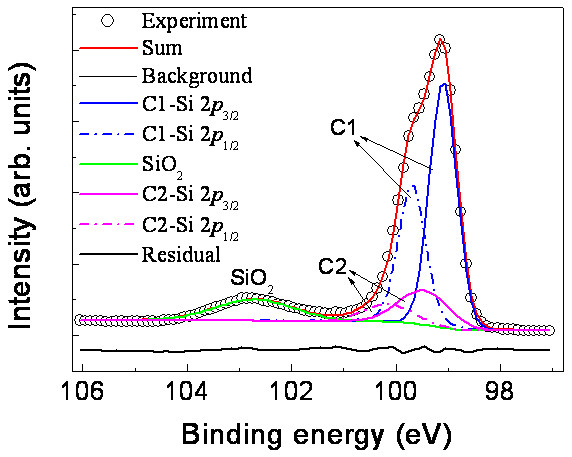
**XPS Si 2*p *spectrum for the *c *(4 × 8) thin film grown on the Si (111) substrate.** The open circles represent the experimental data and the thick solid line (red) overlapping them is the fit to the data. The right side peak can be decomposed into C1 and C2 components. The main component C1 comes from the contribution of Si substrate, while component C2 comes from the contribution of the iron silicide phase. The residual of the fit is shown by the lowermost solid line (black).

Figure 6 shows the Si 2*p* spectrum for the *c* (4 × 8) thin film grown on the Si (111) substrate. The Si 2*p* doublet (Si 2*p*_3/2_ and 2*p*_1/2_) appears at approximately 98 to101 eV but is not well-resolved. The weak and broad peaks at around 102.7 eV can be assigned to the Si 2*p* of SiO_2_. In order to determine the stoichiometry of the *c* (4 × 8) thin film, we performed a curve fitting on the spectrum and the result of the fit is also included in the figure. In the fitting procedure, the spin-orbit splitting was fixed at 0.6 eV for all components. The Si 2*p* spectrum can be decomposed into two components, with the main component C1 at *E*_B_ = 99.2 eV (2*p*_3/2_ line) and the other component C2 at *E*_B_ = 99.5 eV. The C1 component comes from the contribution of Si substrate, while the C2 is associated with the iron silicides formed on the Si substrate. Compared to the bulk Si component, the Si 2*p* peak for the Fe silicides has shifted to a higher binding energy (+0.3 eV) and the FWHM has become wider (+0.4 eV), which is consistent with that reported in the previous studies [21,22]. Quantitative analysis of the XPS data shows that the atomic ratio of Fe/Si in the *c* (4 × 8) thin film is approximately 1:2.05, indicating that the *c* (4 × 8) thin film phase is in the FeSi_2_ stoichiometry regime.

## Conclusions

In summary, using RDE method, we have shown that a homogeneous crystalline iron silicide thin film of *c* (4 × 8) phase can be grown on the Si (111) surface at a temperature above approximately 750°C. The thickness of the *c* (4 × 8) film can be up to approximately 6.3 Å. This result is quite different from the previous results obtained using the SPE method, where the *c* (4 × 8) film has a definite thickness in the range of 1.4 to 1.9 Å. We attribute the larger thickness of the *c* (4 × 8) film obtained by the RDE method to the supply of sufficient free Si atoms during the silicide reaction. Scanning tunneling spectroscopy measurements show that the *c* (4 × 8) thin film exhibits a semiconducting character with a band gap of approximately 0.85 eV. Quantitative XPS analysis shows that the *c* (4 × 8) phase is in the FeSi_2_ stoichiometry regime. This homogeneous *c* (4 × 8) thin film could be used in the optoelectronic devices or serve as a precursor surface applicable in magnetic technological fields.

## Competing interests

The authors declare that they have no competing interests.

## Authors’ contributions

ZQZ designed the project of the experiments and drafted the manuscript. LMS carried out the XPS measurements. GMS, XYL, and XL carried out the growth of the iron silicide thin films and STM measurements. All authors read and approved the final manuscript.
